# Cytokine mRNA Degradation in Cardiomyocytes Restrains Sterile Inflammation in Pressure-Overloaded Hearts

**DOI:** 10.1161/CIRCULATIONAHA.119.044582

**Published:** 2020-01-14

**Authors:** Shigemiki Omiya, Yosuke Omori, Manabu Taneike, Tomokazu Murakawa, Jumpei Ito, Yohei Tanada, Kazuhiko Nishida, Osamu Yamaguchi, Takashi Satoh, Ajay M. Shah, Shizuo Akira, Kinya Otsu

**Affiliations:** 1 The School of Cardiovascular Medicine and Sciences, King’s College London British Heart Foundation Centre of Excellence, United Kingdom (S.O., Y.O., M.T., T.M., J.I., Y.T., K.N., A.M.S., K.O.).; 2 Department of Cardiovascular Medicine, Graduate School of Medicine (O.Y.), Osaka University, Suita, Japan.; 3 Laboratory of Host Defense, Research Institute for Microbial Diseases (T.S., S.A.), Osaka University, Suita, Japan.

**Keywords:** heart failure, inflammation, interleukin-6, RNA stability

## Abstract

**Background:**

Proinflammatory cytokines play an important role in the pathogenesis of heart failure. The mechanisms responsible for maintaining sterile inflammation within failing hearts remain poorly defined. Although transcriptional control is important for proinflammatory cytokine gene expression, the stability of mRNA also contributes to the kinetics of immune responses. Regnase-1 is an RNase involved in the degradation of a set of proinflammatory cytokine mRNAs in immune cells. The role of Regnase-1 in nonimmune cells such as cardiomyocytes remains to be elucidated.

**Methods:**

To examine the role of proinflammatory cytokine degradation by Regnase-1 in cardiomyocytes, cardiomyocyte-specific Regnase-1–deficient mice were generated. The mice were subjected to pressure overload by means of transverse aortic constriction to induce heart failure. Cardiac remodeling was assessed by echocardiography as well as histological and molecular analyses 4 weeks after operation. Inflammatory cell infiltration was examined by immunostaining. Interleukin-6 signaling was inhibited by administration with its receptor antibody. Overexpression of Regnase-1 in the heart was performed by adeno-associated viral vector–mediated gene transfer.

**Results:**

Cardiomyocyte-specific Regnase-1–deficient mice showed no cardiac phenotypes under baseline conditions, but exhibited severe inflammation and dilated cardiomyopathy after 4 weeks of pressure overload compared with control littermates. Four weeks after transverse aortic constriction, the *Il6* mRNA level was upregulated, but not other cytokine mRNAs, including tumor necrosis factor–α, in Regnase-1–deficient hearts. Although the *Il6* mRNA level increased 1 week after operation in both Regnase-1–deficient and control hearts, it showed no increase in control hearts 4 weeks after operation. Administration of anti–interleukin-6 receptor antibody attenuated the development of inflammation and cardiomyopathy in cardiomyocyte-specific Regnase-1–deficient mice. In severe pressure overloaded wild-type mouse hearts, sustained induction of *Il6* mRNA was observed, even though the protein level of Regnase-1 increased. Adeno-associated virus 9–mediated cardiomyocyte-targeted gene delivery of Regnase-1 or administration of anti–interleukin-6 receptor antibody attenuated the development of cardiomyopathy induced by severe pressure overload in wild-type mice.

**Conclusions:**

The degradation of cytokine mRNA by Regnase-1 in cardiomyocytes plays an important role in restraining sterile inflammation in failing hearts and the Regnase-1–mediated pathway might be a therapeutic target to treat patients with heart failure.

## Abstract 2

Supplemental Digital Content is available in the text.

**Clinical Perspective**

**What Is New?**

- The degradation of cytokine mRNA by Regnase-1, an RNase, in cardiomyocytes plays an essential role in restraining inflammation in mouse pressure overload–induced failing hearts.
- The major target for Regnase-1–mediated mRNA degradation appears to be interleukin-6 in cardiomyocytes.
- Sustained increase in *Il6* mRNA by deficiency or insufficient upregulation of Regnase-1 in pressure-overloaded hearts promotes cardiac remodeling and inflammation.

**What Are the Clinical Implications?**

- Failure of appropriate induction of Regnase-1 may underlie the persistent and chronic inflammation seen in chronic heart failure.
- Upregulation of Regnase-1 function or interleukin-6 blockade may be a fruitful approach to therapeutic immunomodulation in patients with heart failure with an increased level of interleukin-6.

Heart failure is the leading cause of death in developed countries. Circulating levels of proinflammatory cytokines, including tumor necrosis factor (TNF)–α, are increased in patients with heart failure and related to the severity and prognosis of the disease, although infection with microorganisms is not involved in most cases.^1^ This suggests an important role of sterile inflammation in the pathogenesis of chronic heart failure. However, targeted anti-TNF approaches were negative with respect to primary trial end points or resulted in worsening heart failure or death.^2^ In addition to TNF-α, the proinflammatory cytokines that are elaborated in heart failure include other members of the TNF superfamily, members of the interleukin (IL)–1 family, and IL-6.^1^ The entire scenario of how inflammation occurs in stressed hearts must be elucidated to develop novel and effective treatments for heart failure.

We have reported previously that incomplete degradation of mitochondrial DNA by lysosomal DNase II in cardiomyocytes results in the initiation of inflammation and development of heart failure in a pressure overload–induced mouse heart failure model.^3^ The mechanisms responsible for maintaining inflammatory responses within failing hearts remain poorly defined. Although transcriptional control is a determinant of the kinetics of proinflammatory cytokine gene expression, the stability of the mRNA also has a key function in coordinating immune responses.^4^

Regnase-1 (also known as Zc3h12a and monocyte chemotactic protein-1–induced protein-1) is an RNase that destabilizes a set of mRNAs, including IL-6 and IL-12b, through cleavage of their 3′ untranslated regions in macrophages.^5^ Regnase-1–deficient mice showed augmented serum immunoglobulin levels, autoantibody production, and infiltration of plasma cells to the lung. Macrophages isolated from Regnase-1–deficient mice showed increased production of IL-6 and IL-12p40 but not TNF. Although Regnase-1 is expressed ubiquitously, the role of Regnase-1 in nonimmune cells such as cardiomyocytes has not been fully elucidated.

In this study, we generated cardiomyocyte-specific Regnase-1–deficient mice to elucidate the role of cytokine mRNA degradation in cardiomyocytes during cardiac remodeling. The results of this study indicate that cytokine mRNA degradation by Regnase-1 in cardiomyocytes is important in the maintenance of sterile inflammation and development of heart failure.

## Methods

The data, analytic methods, and study materials are available from the corresponding author to other researchers on reasonable request for purposes of reproducing the results or replicating the procedure.

### Study Approval

All in vivo and in vitro experimental protocols were approved by the King’s College London Ethical Review Process Committee and UK Home Office (project license PPL70/7260) and were performed in accordance with the Guidance on the Operation of the Animals (Scientific Procedures) Act, 1986 (UK Home Office).

### Generation of Cardiomyocyte-Specific Regnase-1–Deficient Mice

Mice bearing a *Regnase-1*^flox^ allele^6^ were crossed with knock-in mice expressing *Cre* recombinase under the control of myosin light chain 2v (*Mlc2v*) promoter^7^ to produce cardiomyocyte-specific Regnase-1–deficient (*Regnase-1*^flox/flox^;*Mlc2v*-*Cre*^+^) mice. All mice used were on the C57BL/6 and SV129 mixed background and were 8- to 12-week-old male Regnase-1–deficient mice. Their littermates were used as controls. Mice were given food and water ad libitum.

### Isolation of Mouse Adult Cardiomyocytes

Adult cardiomyocytes were isolated from 10- to 12-week-old male mice using a Langendorff system and cultured as we reported previously.^3^

### Echocardiography and Transverse Aortic Constriction

A Vevo 2100 system with a 22- to 55-MHz linear transducer (Visual Sonics) was used to perform echocardiography on conscious mice.^8^ Noninvasive measurement of tail blood pressure was also performed on conscious mice using a blood pressure monitor for rats and mice (Muromachi Kikai) as described previously.^8^ The mice underwent thoracic transverse aortic constriction (TAC) and severe TAC (sTAC) using 26- and 27-gauge needles for aortic constriction, respectively.^3^ In TAC, a small piece of a 6-0 silk suture was placed between the innominate and left carotid arteries. Two loose knots were tied around the transverse aorta and a 26-gauge needle was placed parallel to the transverse aorta. The knots were tied quickly against the needle and the needle was removed promptly to yield a 26-gauge stenosis. In sTAC, aortic constriction was performed by tying a 6-0 silk suture against a 27-gauge needle to yield a more severe constriction. Sham surgeries were identical except for the aortic constriction.

### Administration of MR16-1

After TAC operation, *Reg1*^+/+^ and *Reg1*^−/−^ mice received an intraperitoneal injection of 2 mg anti-mouse IL-6 receptor antibody MR16-1 (a gift from Chugai Pharmaceutical Co, Ltd) or 2 mg control immunoglobulin G (IgG; 855951; MP Biomedicals). Then they were injected intraperitoneally once a week with a total of 3 injections with 0.5 mg MR16-1 or IgG.^9^ For the experiment to examine the effect of MR16-1 on cardiac remodeling in wild-type mice, sTAC-operated C57BL/6 mice received weekly injection with 0.15 mg MR16-1 or IgG from 1 week after sTAC.

### Virus Production and Infection

FLAG-tagged Reg1 was cloned into inverted terminal repeats–containing adeno-associated virus (AAV) plasmid harboring the chicken cardiac troponin T promoter (PL-C-PV1967, Penn Vector Core, University of Pennsylvania) after removing enhanced green fluorescent protein (eGFP) sequence. AAV type 9 (AAV9) encoding FLAG-tagged Reganse-1 (Reg1-AAV9) was generated by transient transfection of HEK293 cells using 3 plasmids (the cis inverted terminal repeats–containing plasmid, the transplasmid encoding AAV replicase and capsid genes, and the adenoviral helper plasmid) in Penn Vector Core. As a control, AV-9-PV1967 (eGFP-AAV9; Penn Vector Core) was used. Eight- to ten-week-old C57BL/6 mice subjected to TAC operation were intraperitoneally injected with 1 × 10^11^ vector genomes of Reg1-AAV9 or eGFP-AAV9 1 week before surgery.

### Western Blot Analysis

Total protein homogenates were subjected to Western blot analysis using a monoclonal mouse antibody to GAPDH (G8795; Sigma), a monoclonal rabbit antibody to Regnase-1 (generated by Prof Akira),^10^ a monoclonal rabbit antibody to phospho-STAT3 (Tyr705; 9145S; Cell Signaling), and a monoclonal mouse antibody to STAT3 (124H6; 9139S; Cell Signaling). After incubation with secondary antibody, the blot was developed with an infrared imaging system (Odyssey CLx; LI-COR Biosciences). Image Studio software (LI-COR Biosciences) was used for quantitative analysis to evaluate protein expression levels.

### Histological Analysis

Left ventricle samples were embedded in the OCT compound (Thermo Fisher Scientific Inc) and then immediately frozen in liquid nitrogen.^8^ The samples were sectioned into 6-µm thickness and fixed with acetone. Hematoxylin-eosin staining and Masson trichrome staining (Masson’s Trichrome Stain Kit, Polysciences Inc) were performed on serial sections. For wheat germ agglutinin staining, heart samples were stained with fluorescein isothiocyanate–conjugated lectin (Sigma) to measure the cross-sectional area of cardiomyocytes. Quantitative analyses of fibrosis fraction and cardiomyocyte cross-sectional areas were examined in 5 different areas (magnification ×200) per section and measured using ImageJ software (National Institutes of Health). Terminal deoxynucleotidyl transferase–mediated dUTP-biotin nick end labeling assay was performed using an in situ apoptosis detection kit (Takara Bio Inc). The number of terminal deoxynucleotidyl transferase–mediated dUTP-biotin nick end labeling–positive nuclei and total nuclei was counted. For immunohistochemical staining, avidin-peroxidase (Vectastain Elite ABC Kit; Vector Laboratories Inc) and DAB Peroxidase Substrate Kit (Vector Laboratories Inc) were used, followed by counterstaining with hematoxylin, as described previously.^8^ Quantitative analyses of inflammatory cells were examined by counting the number of immunopositive cells in 5 different areas (magnification ×200) per section and expressed as the number per millimeters squared.^8^ For quantification in histology, 2 serial heart sections were prepared, and then 3 different areas from the midportion of the free wall and 2 areas from the midportion of the septal wall in each section were assessed. Images were analyzed in a blinded fashion by 2 reviewers. The primary antibodies used were rat anti-CD45 (MAB114; R&D Systems Inc), rat anti-CD68 (MCA1957GA; AbD Serotec) or rabbit anti-CD68 antibody for immunofluorescence (ab125212; Abcam), rat anti-Ly6G/6C (550291; BD Biosciences), rabbit anti-CD3 (ab16669; Abcam), hamster anti-mouse CD11c antibody (MCA1369; Bio-Rad Laboratories Inc), and rat anti-mouse CD206 antibody (MCA2235; Bio-Rad Laboratories Inc). The secondary antibodies were goat anti-rabbit IgG (H+L) highly cross-adsorbed secondary antibody, Alexa Fluor 568 (A-11036; Thermo Fisher Scientific Inc); goat anti-hamster IgG (H+L) secondary antibody, Alexa Fluor 488 (A21110; Thermo Fisher Scientific Inc); and goat anti-rat IgG (H+L) cross-adsorbed secondary antibody, Alexa Fluor 488 (A11006; Thermo Fisher Scientific Inc). DAPI (ProLong Gold Antifade Reagent with DAPI; Life Technologies) was used to detect nuclei.

### Real-Time Quantitative Reverse Transcription Polymerase Chain Reaction

Total RNA was isolated from the left ventricles or isolated cardiomyocytes using TRIzol reagent (Thermo Fisher Scientific Inc). The mRNA expression levels were determined by quantitative reverse transcription polymerase chain reaction (PCR) using SuperScript II Reverse Transcriptase (Thermo Fisher Scientific Inc) for reverse transcription and a Power SYBR Green PCR Master Mix (Thermo Fisher Scientific Inc) for quantitative reverse transcription PCR reaction with PCR primers designed as follows: forward 5′-GAGTGGAAACGCTTCATCGAG-3′ and reverse 5′-AGGAAGTTGTCCAGGCTAGG-3′ for Regnase-1 (*Reg1*), forward 5′-ACAACCACGGCCTTCCCTACTT-3′ and reverse 5′-CACGATTTCCCAGAGAACATGTG-3′ for IL-6 (*Il6*), forward 5′-TCCCAGGTTCTCTTCAAGGGA-3′ and reverse 5′-GGTGAGGAGCACGTAGTCGG-3′ for TNF-α (*Tnfa*), forward 5′-AAGAGCTTCAGGCAGGCAGTATCA-3′ and reverse 5′-TAATGGGAACGTCACACACCAGCA-3′ for IL-1-β (*Il1b*), forward 5′-TCGTCTTGGCCTTTTGGCT-3′ and reverse 5′-TCCAGGTGGTCTAGCAGGTTCT-3′ for atrial natriuretic peptides (*Nppa*), forward 5′-AAGTCCTAGCCAGTCTCCAGA-3′ and reverse 5′-GAGCTGTCTCTGGGCCATTTC-3′ for brain natriuretic peptides (*Nppb*), forward 5′-ACGCGGACTCTGTTGCTGCT-3′ and reverse 5′-GCGGGACCCCTTTGTCCACG-3′ for collagen type I α2 (*Co1a2*), forward 5′-CCCGGGTGCTCCTGGACAGA-3′ and reverse 5′-CACCCTGAGGACCAGGCGGA-3′ for collagen type III α1 (*Col3a1*), and forward 5′-ATGACAACTTTGTCAAGCTCATTT-3′ and reverse 5′-GGTCCACCACCCTGTTGCT-3′ for *Gapdh*.^8^ The PCR primers for IL-12b (*Il12b*; Mm01288989_m1), interferon-b1 (*Ifnb1*; Mm 00439552_s1), interferon-γ (*Ifng*; Mm01168134_m1), and IL-10 (*Il10*; Mm01288386_m1) were purchased from Thermo Fisher Scientific Inc. The TaqMan Gene Expression Master Mix (Thermo Fisher Scientific Inc) was used for amplification of *Il12b*, *Ifnb1*, *Ifng*, *and Il10*. PCR standard curves were constructed using the corresponding cDNA and all data were normalized to *Gapdh* mRNA content and are expressed as fold increase over the control group.

### In Situ Hybridization

In situ hybridization was performed using the QuantiGene ViewRNA Chromogenic Signal Amplification Kit (Affymetrix eBioscience), the QuantiGene ViewRNA ISH Tissue 1-Plex Assay Kit (Affymetrix eBioscience), and the ViewRNA Probe Mouse Il6 (Affymetrix eBioscience) according to the manufacturer’s instructions. After frozen tissue slides were fixed in 10% neutral buffered formalin at 4°C for 16 hours, the slides were dehydrated and digested with protease. Hybridization with the probe for *Il6* mRNA was performed, followed by signal amplification and signal detection steps. After washing in phosphate-buffered saline, the slides were incubated with an anti–α-sarcomeric actin (A2172; Sigma) and chicken anti-mouse IgG (H+L) Cross-Adsorbed Secondary Antibody, Alexa Fluor 488 (A-21200; Thermo Fisher Scientific Inc).

### Statistics

Results are shown as mean ± SEM. Paired data were evaluated by the Student *t* test, which was used for 2-group comparison, and 1-way analysis of variance with the Bonferroni post hoc test was used for multiple comparisons. *P*<0.05 was considered statistically significant.

## Results

### Regnase-1–Deficient Mice Had No Cardiac Phenotypes at Baseline

To examine the in vivo role of Regnase-1 in cardiomyocytes, mice with a *Regnase-1*^flox^ allele^6^ were crossed with knock-in mice expressing *Cre* recombinase under the control of *Mlc2v* promoter^7^ to produce *Regnase-1*^flox/flox^;*Mlc2v*-*Cre*^+^ (*Reg1*^−/−^) mice. *Regnase-1*^flox/flox^;*Mlc2v*-*Cre*^−^ (*Reg1*^+/+^) littermates were used as controls. The *Reg1*^−/−^ mice were born at Mendelian frequency and grew to adulthood. In *Reg1*^−/−^ hearts, there was a significant reduction in the protein level of Regnase-1 (Figure IA in the online-only Data Supplement). The deletion of Reg1 was confirmed in isolated cardiomyocytes by quantitative reverse transcription PCR (Figure IB in the online-only Data Supplement). There were no significant differences in physiological and echocardiographic parameters between *Reg1*^+/+^ and *Reg1*^−/−^ mice (Table I in the online-only Data Supplement), indicating that the *Reg1*^−/−^ mice had normal global cardiac structure and function.

### Regnase-1–Deficient Mice Developed Heart Failure in Response to Pressure Overload

**Figure 1.**
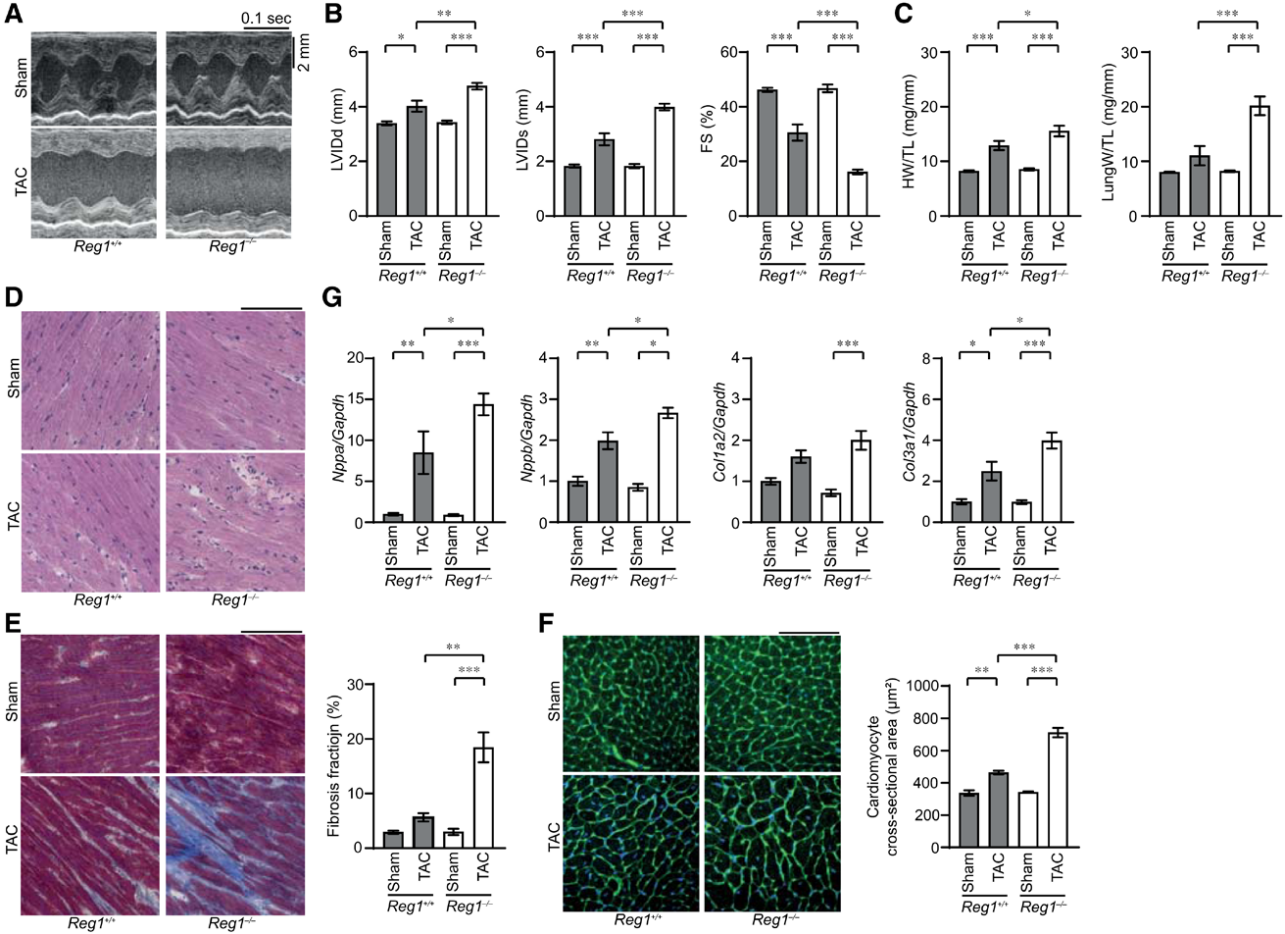
**Pressure overload–induced cardiomyopathy in *Reg1^-/-^* mice.** The *Reg1*^+/+^ and *Reg1*^−/−^ mice were subjected to pressure overload by means of transverse aortic constriction (TAC). The mice were analyzed 4 weeks after TAC. Data were evaluated by 1-way analysis of variance with the Bonferroni post hoc test. Data are mean ± SEM. ^*^P<0.05, ^**^P<0.01, ^***^P<0.001. **A**, M-mode echocardiographic tracings from sham- or TAC-operated *Reg1*^+/+^ or *Reg1*^−/−^ mice. **B** and **C**, Echocardiographic (**B**) and physiologic (**C**) parameters. Total n=7 (sham–*Reg1*^+/+^), 7 (TAC–*Reg1*^+/+^), 7 (sham–*Reg1*^−/−^), or 6 (TAC–*Reg1*^−/−^) per group. **D** through **F**, Hematoxylin-eosin–stained (**D**), Masson trichrome–stained (**E**), and wheat germ agglutinin–stained (**F**) heart sections. Scale bar, 100 µm. Fibrosis fraction was measured (n=3). Cardiomyocyte cross-sectional area was measured by tracing the outline of 70 myocytes in the nonfibrotic area in each section (n=3). **G**, mRNA expression of *Nppa*, *Nppb*, *Col1a2*, and *Col3a1*. Total n=5 (sham–*Reg1*^+/+^), 4 (TAC–*Reg1*^+/+^), 5 (sham–*Reg1*^−/−^), or 5 (TAC–*Reg1*^−/−^) per group. *Gapdh* mRNA was used as the loading control. The averaged value in sham-operated *Reg1*^+/+^ hearts was set equal to 1. FS indicates fractional shortening; HW/TL, heart weight/tibia length; LungW/TL, lung weight/TL; LVIDd, end-diastolic left ventricular internal dimension; and LVIDs, end-systolic left ventricular internal dimension.

To determine the role of Regnase-1 during cardiac remodeling, *Reg1*^+/+^ and *Reg1*^−/−^ mice were subjected to pressure overload by means of TAC.^11^ There was no difference in the survival ratio between TAC-operated *Reg1*^−/−^ mice (8.3%; 1 out of 12 mice) and TAC-operated *Reg1*^+/+^ mice (0%; 0 out of 13 mice). The *Reg1*^+/+^ and *Reg1*^−/−^ mice exhibited left chamber dilation and cardiac dysfunction 4 weeks after TAC (Figure 1A and 1B). The extent was more severe in *Reg1*^−/−^ mice. Although TAC induced an increase in the heart weight to tibia length ratio in both groups, the ratio was larger in *Reg1*^−/−^ mice (Figure 1C). The lung weight to tibia length ratio was elevated in TAC-operated *Reg1*^−/−^ mice, but not in *Reg1*^+/+^ mice (Figure 1C). TAC-operated *Reg1*^−/−^ hearts exhibited intermuscular cell infiltration (Figure 1D). Interstitial fibrosis was present in TAC-operated *Reg1*^−/−^ hearts (Figure 1E). The cardiomyocyte cross-sectional area in TAC-operated *Reg1*^−/−^ mice was larger than in TAC-operated *Reg1*^+/+^ mice (Figure 1F). The mRNA levels of *Nppa* and *Nppb* increased in both TAC-operated *Reg1*^+/+^ and *Reg1*^−/−^ hearts, but were higher in TAC-operated *Reg1*^−/−^ hearts (Figure 1G). The mRNA expression of *Col3a1* in TAC-operated *Reg1*^−/−^ hearts was higher than in TAC-operated *Reg1*^+/+^ hearts (Figure 1G). The number of apoptotic cardiomyocytes increased in TAC-operated *Reg1*^−/−^ hearts (Figure IIA and IIB in the online-only Data Supplement). These data suggest that ablation of Regnase-1 in cardiomyocytes resulted in severe cardiac chamber dilation, dysfunction, hypertrophy, and fibrosis and lung congestion in response to pressure overload.

### Cardiomyocyte-Specific Deletion of Regnase-1 in Pressure-Overloaded Hearts Resulted in the Development of Inflammation With a Specific Increase in *Il6* mRNA

**Figure 2.**
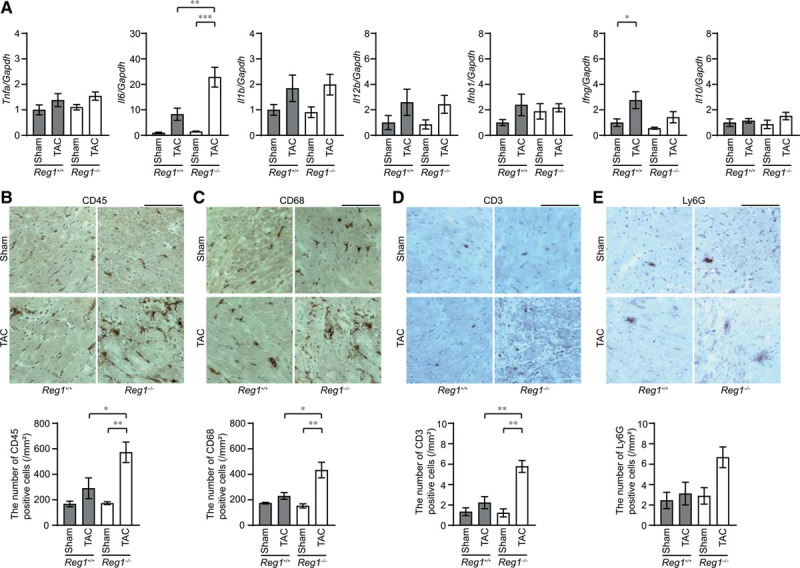
**Inflammatory responses in pressure**-**overloaded Reg1^^-/-^^ hearts.** The *Reg1*^+/+^ and *Reg1*^−/−^ mice subjected to transverse aortic constriction (TAC) were analyzed 4 weeks after TAC. Data were evaluated by 1-way analysis of variance with the Bonferroni post hoc test. Data are mean ± SEM. ^*^P<0.05, ^**^P<0.01, ^***^P<0.001. **A**, Inflammatory cytokine mRNAs including *Tnfa*, *Il6*, *Il1b*, *Il12b*, *Ifnb1*, *Ifng*, and *Il10*. Total n=5 (sham–*Reg1*^+/+^), 4 (TAC–*Reg1*^+/+^), 5 (sham–*Reg1*^−/−^), or 5 (TAC–*Reg1*^−/−^) per group. *Gapdh* mRNA was used as the loading control. The averaged value in sham-operated *Reg1*^+/+^ mice was set equal to 1. **B** through **E**, Immunohistochemical analysis for CD45 (**B**), CD68 (**C**), CD3 (**D**), and Ly6G (**E**). Scale bar, 100 µm. Bottom graphs show quantitative analysis of each infiltrating inflammatory cell type (n=3).

**Figure 3.**
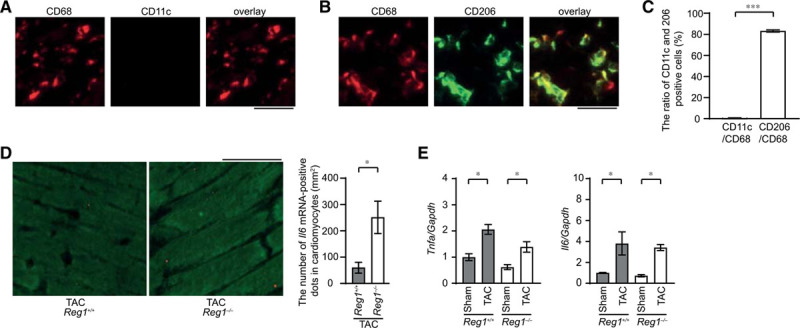
**Production of *I16* mRNA in *Reg1*^-/-^ hearts under pressure overload**. *Reg1*^+/+^ and *Reg1*^−/−^ hearts 4 weeks (**A** through **D**) or 1 week (**E**) after transverse aortic constriction (TAC) were analyzed. Data were evaluated by the Student *t* test (**C** and **D**) or 1-way analysis of variance with the Bonferroni post hoc test (**E**). Data are mean ± SEM. ^*^P<0.05, ^***^P<0.001. **A**, Double staining of TAC-operated *Reg1*^−/−^ heart sections with anti-CD68 (red) and anti-CD11c (green) antibodies. **B**, Double staining of TAC-operated *Reg1*^−/−^ heart sections with anti-CD68 (red) and anti-CD206 (green) antibodies. Scale bar, 100 µm. **C**, The ratio of CD11c-positive or CD206-positive to CD68-postive cell numbers (n=3). **D**, In situ hybridization for *Il6* mRNA (red) in *Reg1*^+/+^ or *Reg1*^−/−^ hearts, followed by immunostaining with α-sarcomeric action antibody (green). Scale bar, 100 µm. Right graph shows the number of red dots in cardiomyocytes. **E**, *Tnfa* and *Il6* mRNA levels 1 week after TAC. Total n=5 (sham–*Reg1*^+/+^), 5 (TAC–*Reg1*^+/+^), 5 (sham–*Reg1*^−/−^), or 6 (TAC–*Reg*1^−/−^) per group. *Gapdh* mRNA was used as the loading control. The averaged value in sham-operated *Reg1*^+/+^ mice was set equal to 1.

Four weeks after TAC, the *Il6* mRNA level was upregulated, but not other cytokine mRNAs, including *Tnfa* and *Il12b* (a known Regnase-1 target),^5^ in TAC-operated *Reg1*^−/−^ hearts compared with the corresponding sham-operated and TAC-operated *Reg1*^+/+^ hearts (Figure 2A). The *Il6* mRNA level was not significantly increased in TAC-operated *Reg1*^+/+^ hearts compared with sham-operated *Reg1*^+/+^ hearts (Figure 2A). A higher number of CD45^+^, CD68^+^, and CD3^+^, but not Ly6G^+^ cells infiltrated TAC-operated *Reg1*^−/−^ hearts than TAC-operated *Reg1*^+/+^ hearts (Figure 2B through 2E). Most inflammatory cells were CD68^+^ macrophages, especially CD206^+^ M2-macrophages (Figure 3A through 3C). The in situ hybridization analysis indicates that a higher number of cardiomyocytes expressed *Il6* mRNA in *Reg1*^−/−^ hearts compared with *Reg1*^+/+^ hearts under pressure overload (Figure 3D). In contrast to the results 4 weeks after TAC, *Il6* and *Tnfa* mRNAs increased in both TAC-operated *Reg1*^+/+^ and *Reg1*^−/−^ hearts and there was no significant difference in the level of the cytokine mRNAs between the 2 groups 1 week after TAC (Figure 3E). Phosphorylation of STAT3, a downstream of IL-6 signaling pathway, was increased in TAC-operated *Reg1*^−/−^ hearts compared with TAC-operated *Reg1*^+/+^ hearts (Figure IIC in the online-only Data Supplement). Thus deficiency of Regnase-1 in cardiomyocytes caused sustained induction of *Il6* mRNA with severe infiltration of inflammatory cells in the heart in response to pressure overload.

### IL-6 Blockade Attenuated Inflammation and Heart Failure in Regnase-1–Deficient Mice

**Figure 4.**
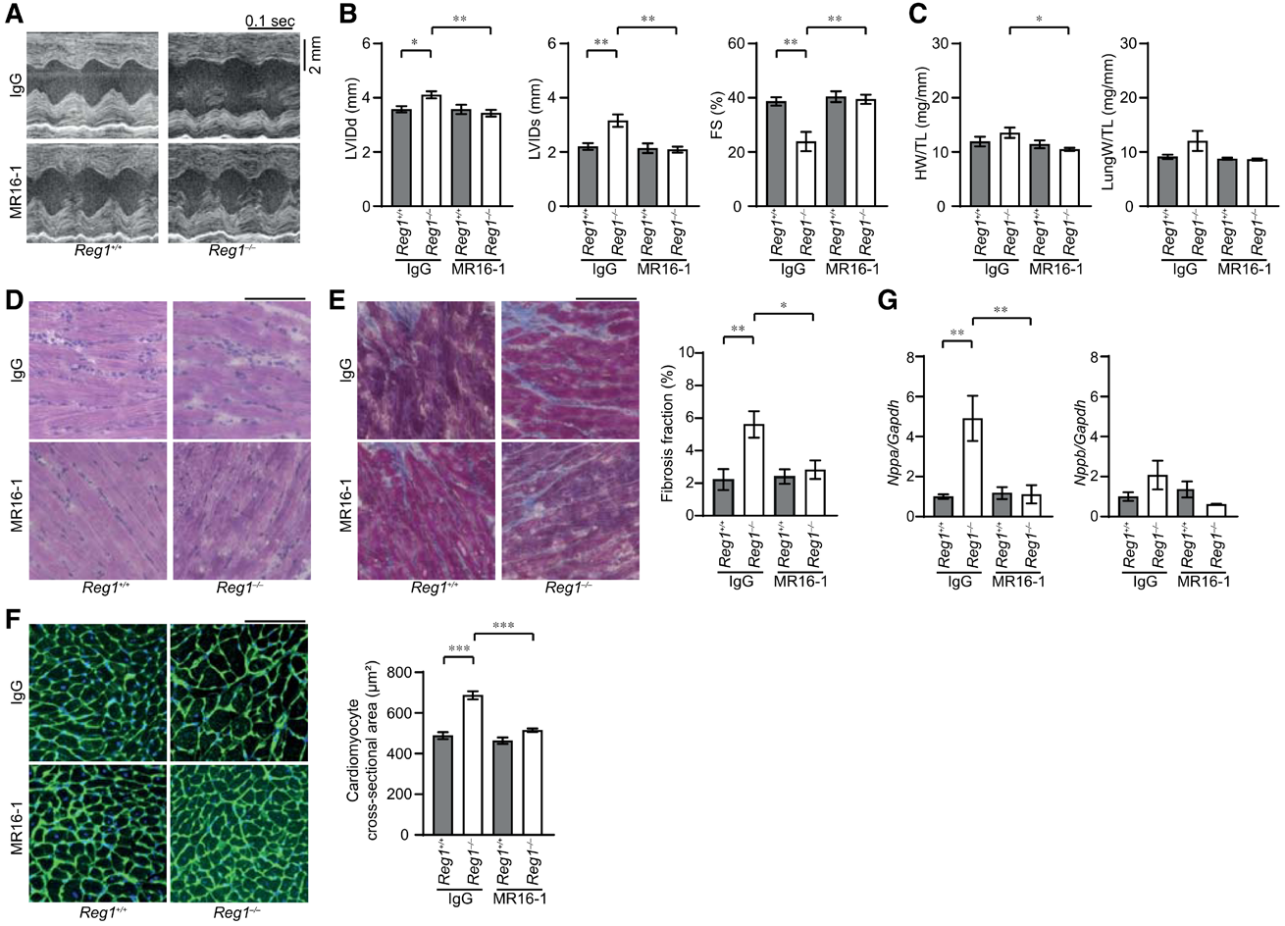
**Interleukin-6 blockade ameliorated transverse aortic constriction–induced cardiomyopathy in *Reg1^-/-^* mice.** After transverse aortic constriction (TAC) operation, *Reg1*^+/+^ and *Reg1*^−/−^ mice received an intraperitoneal injection of anti-mouse interleukin-6 receptor antibody MR16-1 or control immunoglobulin G (IgG). Afterwards, they were injected intraperitoneally once a week with a total of 3 injections with either MR16-1 or IgG. The mice were analyzed 4 weeks after TAC. Data were evaluated by 1-way analysis of variance with the Bonferroni post hoc test. Data are mean ± SEM. ^*^P<0.05, ^**^P<0.01, ^***^P<0.001. **A**, M-mode echocardiographic tracings from IgG-treated or MR16-1-treated *Reg1*^+/+^ or *Reg1*^−/−^ mice. **B** and **C**, Echocardiographic (**B**) and physiologic (**C**) parameters. Total n=7 (IgG–*Reg1*^+/+^), 8 (IgG–*Reg1*^−/−^), 6 (MR16-1–*Reg1*^+/+^), or 8 (MR16-1–*Reg1*^−/−^) per group. **D** through **F**, Hematoxylin-eosin–stained (**D**), Masson trichrome–stained (**E**), and wheat germ agglutinin–stained (**F**) heart sections. Scale bar, 100 µm. Fibrosis fraction (n=5) and cross-sectional area of cardiomyocytes (n=3) were measured. **G**, mRNA expression of *Nppa* and *Nppb*. Total n=6 (IgG–*Reg1*^+/+^), 5 (IgG–*Reg1*^−/−^), 5 (MR16-1–*Reg*1^+/+^), or 5 (MR16-1–*Reg1*^−/−^) per group. *Gapdh* mRNA was used as the loading control. The averaged value in TAC-operated *Reg1*^+/+^ hearts treated with IgG was set equal to 1. FS indicates fractional shortening; HW/TL, heart weight/tibia length; LungW/TL, lung weight/TL; LVIDd, end-diastolic left ventricular internal dimension; and LVIDs, end-systolic left ventricular internal dimension.

**Figure 5.**
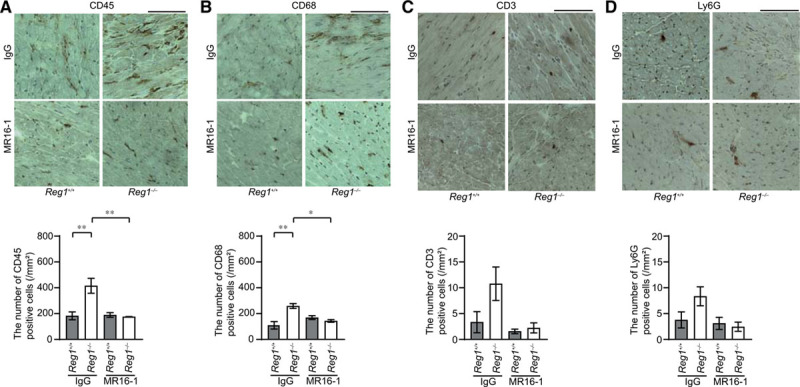
**Interleukin-6 blockade inhibited infiltration of inflammatory cells.** Transverse aortic constriction (TAC)–operated *Reg1*^+/+^ and *Reg1*^−/−^ hearts treated with anti-mouse interleukin-6 receptor antibody MR16-1 or control immunoglobulin G (IgG) were analyzed. Data were evaluated by 1-way analysis of variance with the Bonferroni post hoc test. Data are mean ± SEM. ^*^P<0.05, ^**^P<0.01. **A** through **D**, Immunohistochemical analysis for CD45 (**A**), CD68 (**B**), CD3 (**C**), and Ly6G (**D**). Scale bar, 100 µm. Bottom graphs show quantitative analysis of each infiltrating inflammatory cell type (n=3).

To examine whether the persistent elevation of *Il6* mRNA is a cause for pressure overload–induced heart failure in *Reg1*^−/−^ mice, IL-6 signaling was blocked using a monoclonal antibody against the IL-6 receptor (MR16-1) after TAC surgery.^9^ Control IgG or MR16-1 had no effect on cardiac chamber size and function and heart and lung weight in sham-operated *Reg1*^+/+^ and *Reg1*^−/−^ mice (Figure IIE and IIF in the online-only Data Supplement). MR16-1 attenuated the chamber dilation, cardiac dysfunction, and hypertrophy induced by TAC in *Reg1*^−/−^ mice (Figure 4A through 4C). Furthermore, MR16-1 attenuated noncardiomyocyte cell infiltration, fibrosis, increase in cardiomyocyte cross-sectional area, upregulation of *Nppa* mRNA, and increase in number of apoptotic cardiomyocytes (Figure 4D through 4G and Figure IID in the online-only Data Supplement). Infiltration of CD45^+^ and CD68^+^ cells was also inhibited by MR16-1 (Figure 5A through 5D). In contrast, MR16-1 had no beneficial effect on cardiac abnormalities observed in *Reg1*^+/+^ mice, which exhibited no increase in *Il6* mRNA 4 weeks after TAC (Figure 4A through 4C).

### Severe Pressure Overload Induced Sustained *Il6* mRNA Upregulation in Hearts

Because the plasma level of IL-6 in the patients with heart failure was related to its severity,^12^ severe pressure overload (sTAC) may increase the level of *Il6* mRNA in mouse failing hearts. The wild-type C57BL/6 mice showed chamber dilation, cardiac dysfunction, and lung congestion 1 and 4 weeks after sTAC (Figure IIIA and IIIB in the online-only Data Supplement). Noncardiomyocyte infiltration, fibrosis, infiltration of CD45^+^ and CD68^+^ cells, and upregulation of *Nppa*, *Nppb*, *Col1a2*, and *Col3a1* mRNA were observed 4 weeks after sTAC (Figure IIIC and IIID in the online-only Data Supplement). The levels of *Il6* and *Tnfa* mRNA in the hearts increased 1 week after sTAC compared with those in sham-operated hearts and the level of *Il1b* mRNA was not different between sTAC- and sham-operated hearts 1 week after surgery (Figure IIIE through IIIG in the online-only Data Supplement). The level of *Il6* mRNA was higher in sTAC-operated hearts than in sham-operated hearts 4 weeks after sTAC, whereas the levels of *Tnfa* and *Il1b* mRNA showed no difference between sTAC- and sham-operated hearts 4 weeks after surgery (Figure IIIE through IIIG in the online-only Data Supplement). Thus we switched to a sTAC model to examine the effect of overexpression of Regnase-1 or administration of MR16-1 in wild-type mice, in which *Il6* mRNA was upregulated.

*Reg1*^−/−^ mice exhibited more severe left chamber dilation and cardiac dysfunction 4 weeks after sTAC compared with *Reg1*^+/+^ mice (Figure IIIH and IIII in the online-only Data Supplement) as observed in TAC-operated *Reg1*^+/+^ and *Reg1*^−/−^ mice.

### Regnase-1 Overexpression or IL-6 Blockade in Wild-Type Hearts Attenuated Heart Failure

**Figure 6.**
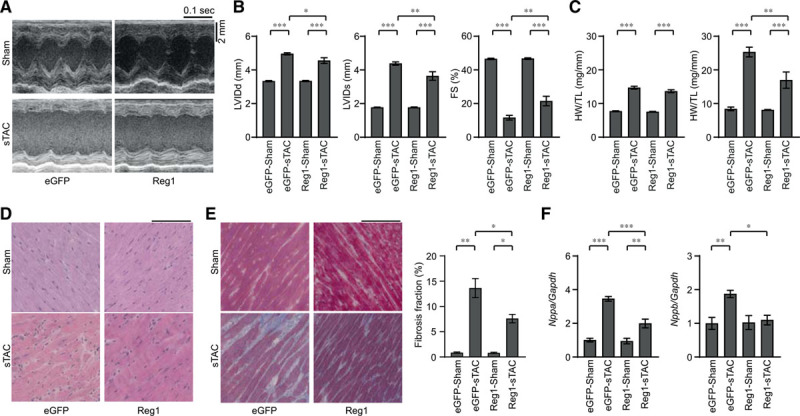
**Overexpression of Regnase-1 protein attenuated severe transverse aortic constriction–induced heart failure.** Wild-type C57BL/6 mice were intraperitoneally injected with adeno-associated virus expressing enhanced green fluorescent protein (eGFP-AAV9) or Regnase-1 (Reg1-AAV9) and were subjected to severe transverse aortic constriction (sTAC) 1 week after infection. Sham- or sTAC-operated wild-type mice infected with eGFP-AAV9 (eGFP–sham or eGFP–sTAC) or Reg1-AAV9 (Reg1–sham or Reg1–sTAC) were analyzed 4 weeks after operation. Data were evaluated by 1-way analysis of variance with the Bonferroni post hoc test. Data are mean ± SEM. ^*^P<0.05, ^**^P<0.01, ^***^P<0.001. **A**, M-mode echocardiographic tracings from eGFP–sham, eGFP–sTAC, Reg1–sham, or Reg1–sTAC wild-type mice. **B** and **C**, Echocardiographic (**B**) and physiologic (**C**) parameters. Total n=16 (eGFP–sham), 11 (eGFP–sTAC), 16 (Reg1–sham), or 15 (Reg1–sTAC). **D** and **E**, Hematoxylin-eosin–stained (**D**) and Masson trichrome–stained (**E**) heart sections. Scale bar, 100 µm. Fibrosis fraction was evaluated. Total n=3 (eGFP–sham or Reg1–sham) or 4 (eGFP–sTAC or Reg1–sTAC). **F**, mRNA expressions of *Nppa* and *Nppb*. Total n=7 (eGFP–sham), 6 (eGFP–sTAC), 7 (Reg1–sham), or 7 (Reg1–sTAC). *Gapdh* mRNA was used as the loading control. The averaged value in the eGFP–sham group was set equal to 1. FS indicates fractional shortening; HW/TL, heart weight/tibia length; LungW/TL, lung weight/TL; LVIDd, end-diastolic left ventricular internal dimension; and LVIDs, end-systolic left ventricular internal dimension.

**Figure 7.**
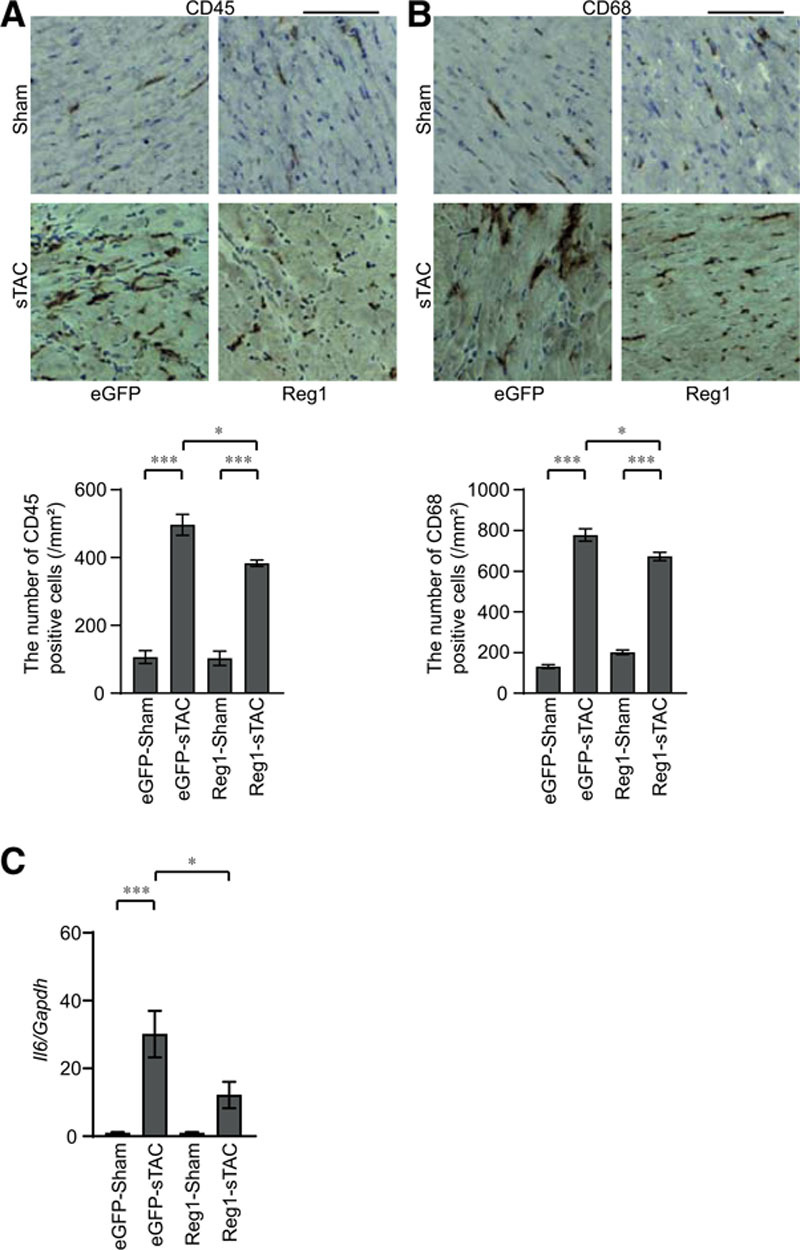
**Induction of Regnase-1 protein suppressed the extent of inflammatory responses in severe transverse aortic constriction–induced heart failure.** Severe transverse aortic constriction (sTAC)–operated wild-type C57BL/6 mice infected with adeno-associated virus expressing enhanced green fluorescent protein(eGFP–sTAC) or Regnase-1 (Reg1–sTAC) were analyzed. Data were evaluated by 1-way analysis of variance with the Bonferroni post hoc test. Data are mean ± SEM. ^*^P<0.05, ^***^P<0.001. **A** and **B**, Immunohistochemical analysis for CD45 (**A**) and CD68 (**B**). Scale bar, 100 µm. Bottom graphs show quantitative analysis of each infiltrating inflammatory cell type in eGFP–sham, eGFP–sTAC, Reg1–sham, or Reg1–sTAC hearts (n=3). **C**, *Il6* mRNA expressions. Total n=7 (eGFP–sham), 6 (eGFP–sTAC), 7 (Reg1–sham), or 7 (Reg1–sTAC). *Gapdh* mRNA was used as the loading control. The averaged value in the eGFP–sham group was set equal to 1.

Upper and lower bands on Western blot represent phosphorylated and nonphosphorylated Regnase-1^10^ in sTAC-operated wild-type C57BL/6 hearts 4 weeks after surgery (Figure IIIJ in the online-only Data Supplement), respectively, both of which exhibited reduced density in *Reg1*^−/−^ hearts (Figure IA in the online-only Data Supplement). Protein levels of nonphosphorylated Regnase-1 and total Regnase-1 were significantly increased in sTAC-operated mouse hearts, but there was no significant difference in phosphorylated Regnase-1 between sham- and sTAC-operated hearts (Figure IIIJ in the online-only Data Supplement). To test whether insufficient induction of Regnase-1 during cardiac remodeling may lead to sustained upregulation of *Il6* mRNA, Reganse-1 was overexpressed in wild-type mouse cardiomyocytes by infection of recombinant Reg1-AAV9 under the control of cardiac troponin T promoter. One week after intraperitoneal injection with AAV9 expressing Regnase-1 or eGFP (eGFP-AAV9), wild-type mice were subjected to sTAC. There were no significant differences in cardiac function 1 week after the infection between the 2 groups (Table II in the online-only Data Supplement). The mice were observed for 4 weeks after surgery. Infection of Reg1-AAV9 resulted in 8.4-fold increase in Regnase-1 protein level in the hearts compared with controls infected with eGFP-AAV9 (Figure IVA in the online-only Data Supplement). There was no significant difference in mortality in Reg1-AAV9-infected mice (21.1%; 4 out of 19 mice) versus that in control vector–infected mice (21.4%; 3 out of 14 mice). Echocardiography revealed improvement in cardiac chamber dilation and function in Reg1-AAV9-infected mice compared with control vector–infected mice (Figure 6A and 6B). Regnase-1 overexpression reduced the ratio of lung weight to tibia length (Figure 6C) and attenuated noncardiomyocyte cell infiltration, fibrosis, upregulation of *Nppa* and *Nppb* mRNA (Figure 6D through 6F), and infiltration of CD45^+^ and CD68^+^ cells (Figure 7A and 7B), and decreased *Il6* mRNA level (Figure 7C) in sTAC-operated hearts. Interestingly, overexpression of Regnase-1 did not show beneficial effects on left chamber dilation and cardiac dysfunction induced by TAC in wild-type mice, in which the level of *Il6* mRNA was not significantly increased (Figure IVB through IVD in the online-only Data Supplement).

Next, we examined the effect of MR16-1 on cardiac remodeling after sTAC in wild-type mice. Because IgG (0.5 mg) administration seems to have a nonspecific cardioprotective effect (Figures 1 and 4), we examined the dose-dependent effect of IgG on cardiac remodeling. C57BL/6 mice were subjected to sTAC operation and received intraperitoneal injection of various doses of IgG (0, 0.15, or 0.50 mg) once a week from 1 week after operation. No effect of 0.15 mg IgG on left chamber dilation and cardiac dysfunction was noted, whereas 0.5 mg IgG attenuated the development of cardiac remodeling (Figure VA in the online-only Data Supplement). Thus 0.15 mg IgG or MR16-1 were injected into the mice to examine the effect of IL-6 blockade on cardiac remodeling. MR16-1 attenuated left ventricular dilation, cardiac dysfunction, hypertrophy, lung congestion, and fibrosis induced by sTAC in wild-type mice (Figure VB through VD in the online-only Data Supplement).

## Discussion

Our data indicate that during normal embryonic development, there is no cardiac myocyte–autonomous requirement for the Regnase-1 signaling pathway. Furthermore, the Regnase-1–mediated pathway does not appear to be required for normal heart growth in the postnatal period. In response to pressure overload, Regnase-1 plays a protective role against the development of heart failure.

In *Reg1*^−/−^ mice, *Il6* mRNA levels increased 1 and 4 weeks after TAC, whereas in *Reg1*^+/+^ mice, the cytokine mRNA was upregulated 1 week after TAC, but not 4 weeks after TAC. *Il6* mRNA is known to be a target of Regnase-1.^5^ Thus *Il6* mRNA degradation by Regnase-1 in cardiomyocytes regulates the time course of its expression level in the heart. Protective effects of MR16-1 in *Reg1*^−/−^ mice indicate that the observed cardiac phenotypes in *Reg1*^−/−^ mice are, at least in part, attributable to the continuous elevation of *Il6* mRNA. However, the lack of beneficial effect of MR16-1 on cardiac contractility in *Reg1*^+/+^ mice indicates that cardiac dysfunction observed in *Reg1*^+/+^ mice is IL-6-independent. Inhibition of IL-6 reduced fibrosis and apoptosis in TAC-operated *Reg1*^−/−^ mice and overexpression of Regnase-1 and inhibition of IL-6 reduced fibrosis in sTAC-operated wild-type mice, suggesting that loss of cardiomyocytes is involved in IL-6-mediated cardiac dysfunction. It has been reported that IL-6 decreases cardiac contractility by the STAT3–nitric oxide–dependent pathway.^13^ We observed increased activation of STAT3 in TAC-operated *Reg1*^−/−^ mice, indicating that the negative inotropic effect of IL-6 may also be involved in the pathogenesis.

No significant increase in *Il12b* mRNA (a known Regnase-1 target) was observed in *Reg1*^+/+^ and *Reg1*^−/−^ hearts 4 weeks after TAC. The major target for Regnase-1–mediated mRNA degradation appears to be IL-6 in cardiomyocytes. In T cells, Regnase-1 regulates the production of interferon-γ mRNA, whereas in macrophages, it regulates the degradation of *Il6* and *Il12p40* mRNAs.^5^ The target of Regnase-1 seems to be cell type–specific.

Our data showed that the time course of *Il6* mRNA level during cardiac remodeling depends on the strength of the stress. This is in agreement with a clinical study that showed that the plasma level of IL-6 in patients with heart failure was related to its severity.^12^ To understand the role of myocardial Regnase-1 upregulation during cardiac remodeling, we used the severe TAC model, in which *Il6* mRNA showed continuous upregulation until 4 weeks after operation. Overexpression of Regnase-1 in cardiomyocytes decreased *Il6* mRNA level in the heart and attenuated the development of myocardial inflammation and heart failure in a sTAC-operated wild-type mouse model. Thus upregulation of Regnase-1 is a mechanism to protect hearts against pressure overload and the level of its upregulation is insufficient to suppress the development of inflammation and dilated cardiomyopathy. MR16-1 attenuated the development of cardiac remodeling in sTAC-operated wild-type mice. Targeting IL-6 might be a fruitful treatment for patients with a high level of IL-6. Cytokine mRNA degradation in cardiomyocytes may be a new potential target for heart failure therapy.

Regnase-1 was reported to be monocyte chemotactic protein-1–induced protein-1.^14^ Monocyte chemotactic protein-1 is the main chemotactic factor for migration of monocytes/macrophages and the pathogenesis of chronic inflammation.^15^ Cardiomyocyte-targeted expression of monocyte chemotactic protein-1 in mice resulted in the induction of monocyte chemotactic protein-1–induced protein-1 and development of cardiac dysfunction with an increased number of apoptotic cardiomyocytes.^14^ However, the present study shows that Reganase-1 protects the heart against hemodynamic stress, inconsistent with these reports showing the detrimental role of monocyte chemotactic protein-1–monocyte chemotactic protein-1–induced protein-1 pathways. Excessive overexpression of Regnase-1 from the embryonic stage might be detrimental to the heart.

The data suggest that the degradation of cytokine mRNA, as well as mitochondrial DNA, in nonimmune cardiomyocytes is critical for restraining inflammation in failing hearts. The Regnase-1-related signaling pathway in cardiomyocytes is a potential therapeutic target to treat patients with heart failure.

## Acknowledgments

The authors thank Brodie Quine, Darran Hardy, Dr Erika Cadoni, and Dr Saki Nakagawa for technical assistance.

## Sources of Funding

Supported by British Heart Foundation (CH/11/3/29051 and RG/16/15/32294), European Research Council (692659), and Japan Society for the Promotion of Science KAKENHI (18H02807) grants to Dr Otsu.

## Disclosures

None.

## Supplementary Material


