# Supplementary material for: Cytokine mRNA Degradation in Cardiomyocytes Restrains Sterile Inflammation in Pressure-Overloaded Hearts
Source: Circulation. 2020 Jan 14;141(8):667–77. doi: 10.1161/CIRCULATIONAHA.119.044582 (PMC7034406; doi:10.1161/CIRCULATIONAHA.119.044582)
Supplement: Supplementary file 1 [file cir-141-667-s001.pdf]

SUPPLEMENTAL MATERIAL

Supplemental Table I. Physiological and echocardiographic parameters in 10- to 12-week-old *Reg1*$^{+/+}$ and *Reg1*$^{-/-}$ mice at baseline.

	Reg1$^{+/+}$ (N=6)	*Reg1*$^{-/-}$ (N=5)
Body weight (g)	28.5 ± 0.8	28.8 ± 1.2
Blood pressure (mmHg)	106 ± 6	112 ± 3
Heart rate (bpm)	726 ± 10	719 ± 8
Heart weight / TL (mg/mm)	8.5 ± 0.2	9.1 ± 0.3
Lung weight / TL (mg/mm)	8.7 ± 0.2	8.7 ± 0.3
Liver weight / TL (mg/mm)	85.6 ± 3.4	77.3 ± 7.5
TL (mm)	17.1 ± 0.1	17.1 ± 0.2
IVSd (mm)	0.77 ± 0.02	0.78 ± 0.02
LVIDd (mm)	3.27 ± 0.08	3.20 ± 0.06
LVIDs (mm)	1.72 ± 0.07	1.69 ± 0.04
LVPWd (mm)	0.75 ± 0.02	0.78 ± 0.02
FS (%)	47.57 ± 1.30	47.22 ± 0.71

TL, tibia length; IVSd, end-diastolic interventricular septum thickness; LVIDd, end-diastolic left ventricular internal dimension; LVIDs, end-systolic left ventricular internal dimension; LVPWd, end-diastolic left ventricular posterior wall thickness; FS, fractional shortening. Data are mean ± SEM. Paired data were evaluated by Student's *t*-test.

Supplemental Table II. Body weight, blood pressure and echocardiographic parameters in 10-week-old C57BL/6 mice 1 week after infection of AAV9 encoding eGFP or Regnase-1.

	eGFP-AAV9 (N=11)	Reg1-AAV9 (N=15)
Body weight (g)	26.2 ± 0.5	26.1 ± 0.4
Blood pressure (mmHg)	121 ± 5	119 ± 4
Heart rate (bpm)	735 ± 4	713 ± 8
IVSd (mm)	0.71 ± 0.01	0.72 ± 0.01
LVIDd (mm)	3.25 ± 0.04	3.19 ± 0.05
LVIDs (mm)	1.71 ± 0.02	1.66 ± 0.05
LVPWd (mm)	0.71 ± 0.01	0.71 ± 0.01
FS (%)	47.22 ± 0.82	48.08 ± 0.83

The C57BL/6 mice infected with AAV9 expressing eGFP (eGFP-AAV9) or Regnase-1 (Reg1-AAV9). IVSd, end-diastolic interventricular septum thickness; LVIDd, end-diastolic left ventricular internal dimension; LVIDs, end-systolic left ventricular internal dimension; LVPWd, end-diastolic left ventricular posterior wall thickness; FS, fractional shortening. Data are mean ± SEM. Paired data were evaluated by Student's *t*-test.

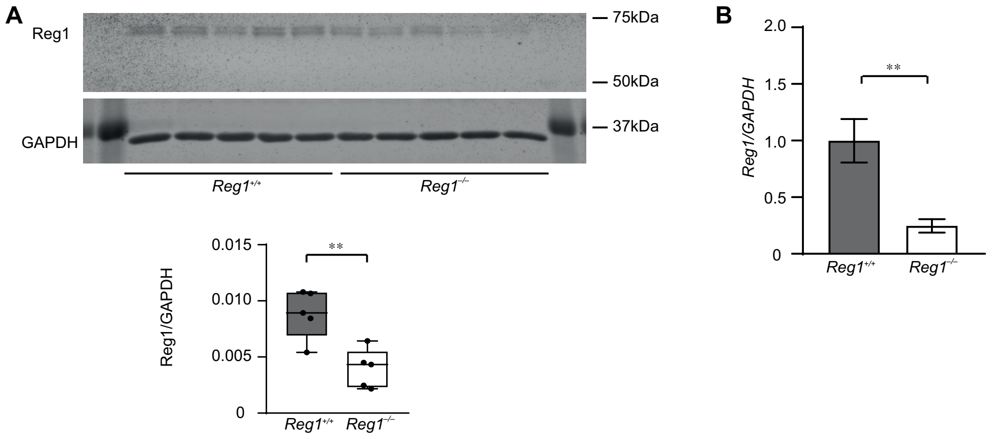

Supplemental Figure I. Regnase-1 expression levels in *Reg1*$^{+/+}$ and *Reg1*$^{-/-}$ hearts.
Data are mean ± SEM. $^{**}P< 0.01$. **A,** Western blot analysis of Regnase-1 (Reg1) protein in hearts (N=5). GAPDH was used as the loading control. The bottom graph shows densitometric analysis. **B,** mRNA expression of *Regnase-1* in cardiomyocytes. The averaged value for *Reg1*$^{+/+}$ cardiomyocytes was set equal to 1. N=6 (*Reg1*$^{+/+}$) or N=7 (*Reg1*$^{-/-}$). *Gapdh* mRNA was used as the loading control.

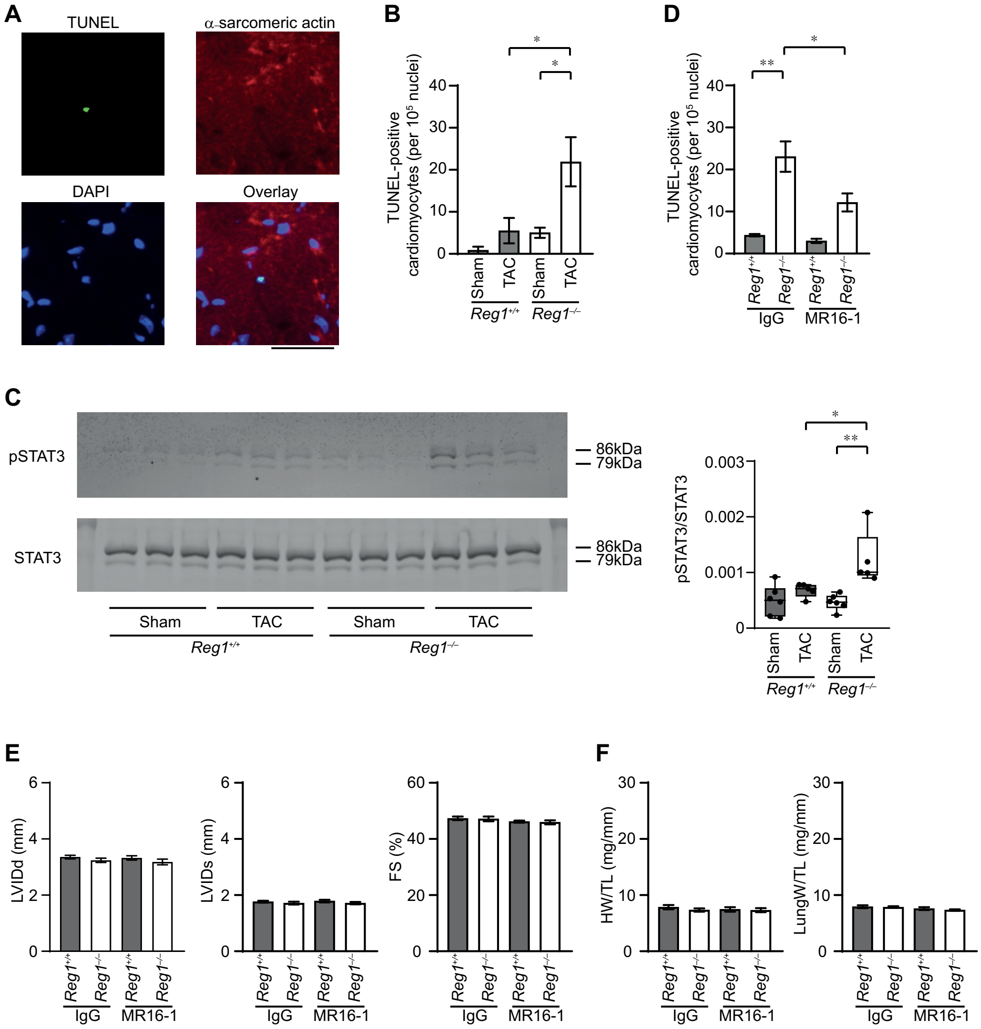

Supplemental Figure II. Apoptotic cardiomyocyte cell death in *Reg1*$^{-/-}$ hearts 4 weeks after TAC and effect of IL-6 blockade using the monoclonal anti-IL6 receptor antibody on apoptosis in *Reg1*$^{-/-}$ hearts 4 weeks after TAC.

Data are mean ± SEM. $^{*}P<0.05$, $^{**}P<0.01$. **A,** Images of apoptotic cardiomyocytes. Triple staining of mouse hearts with DAPI (blue), anti-α-sarcomeric actin antibody (red) and TUNEL (green). Overlay image shows a TUNEL-positive nucleus in a cardiomyocyte. Scale bar, 100 µm. **B,** The number of TUNEL-positive cardiomyocytes in sham- or TAC-operated *Reg1*$^{+/+}$ or *Reg1*$^{-/-}$ mice (N=3). **C,** Protein expression levels of phosphorylated and total STAT3 in sham- or TAC-operated *Reg1*$^{+/+}$ or *Reg1*$^{-/-}$ mice. N=6 (sham-*Reg1*$^{+/+}$), 5 (TAC-*Reg1*$^{+/+}$), 6 (sham-*Reg1*$^{-/-}$) or 5 (TAC-*Reg*1$^{-/-}$) per group. The right graph shows densitometric analysis. **D,** The number of TUNEL-positive cardiomyocytes in TAC-operated *Reg1*$^{+/+}$ or *Reg1*$^{-/-}$ mice treated with anti-mouse IL-6 receptor antibody MR16-1 or control IgG (N=3). **E and F,** After sham operation, *Reg1*$^{+/+}$ and *Reg1*$^{-/-}$ mice received an intraperitoneal injection of anti-mouse IL-6 receptor antibody MR16-1 or control IgG. Afterwards they were injected intraperitoneally once a week with a total of three injections with either MR16-1 or IgG. The mice were analyzed 4 weeks after surgery. Data were evaluated by one-way ANOVA with the Bonferroni's post hoc test. Data are mean ± SEM. $^{*}P<0.05$. Echocardiographic (**E**) and physiological (**F**) parameters. N=6 (IgG-*Reg1*$^{+/+}$), 5 (IgG-*Reg*1$^{-/-}$), 6 (MR16-1-*Reg1*$^{+/+}$) or 6 (MR16-1-*Reg1*$^{-/-}$) per group.

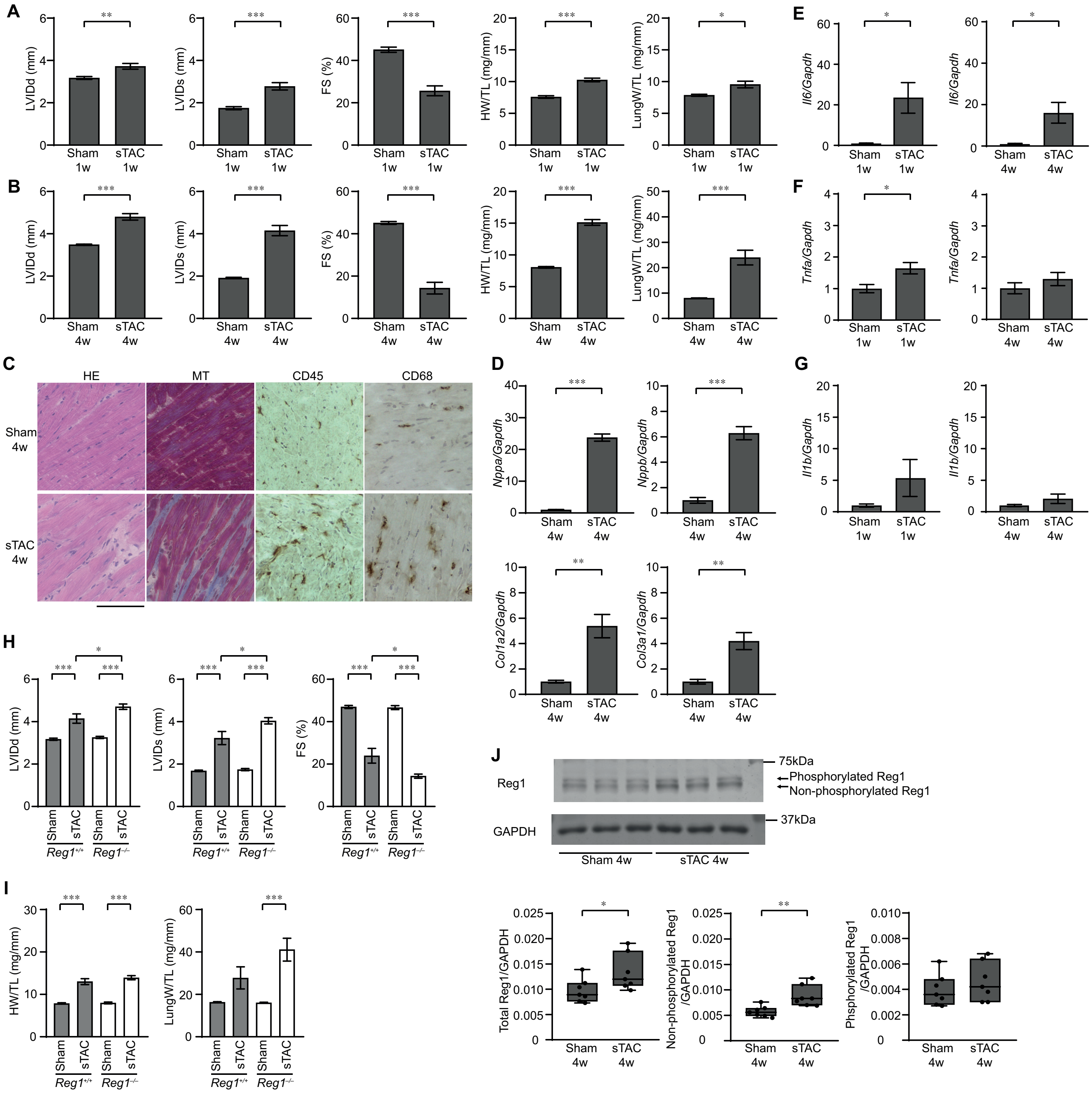

Supplemental Figure III. Cardiac phenotypes of C57BL/6 mice subjected to severe TAC. Data are mean ± SEM. $^{*}P<0.05$, $^{**}P<0.01$, $^{***}P<0.001$. **A** and **B,** Echocardiographic and physiological parameters in C57BL/6 mice 1 (**A**) and 4 (**B**) weeks after severe TAC (sTAC). N=8 (sham 1w), 6 (sTAC 1w), or 12 (4w) per group. **C,** Representative images of hematoxylin-eosin-staining, Masson's trichrome staining and immunohistochemical analysis using antibodies to CD45 and CD68 4 weeks after sTAC. Scale bar, 100 µm. **D,** mRNA expressions of *Nppa*, *Nppb*, *Col1a2,* and *Col3a1* 4 weeks after sTAC (N=5). **E**–**G,** mRNA expressions of inflammatory cytokines such as *Il6* (**E**), *Tnfa* (**F**), and *Il1b* (**G**) 1 or 4 weeks after sTAC (N=5). *Gapdh* mRNA was used as the loading control. The averaged value in sham-operated C57BL/6 mice was set equal to 1. **H** and **I,** The *Reg1*$^{+/+}$ and *Reg1*$^{-/-}$ mice were subjected to severe TAC (sTAC). The mice were analyzed 4 weeks after sTAC. Data were evaluated by one-way analysis of variance (ANOVA) with the Bonferroni's post hoc test. Data are mean ± SEM. $^{*}P < 0.05$. Echocardiographic (**H**) and physiological (**I**) parameters. N=10 (sham-*Reg1*$^{+/+}$), 11 (sTAC-*Reg1*$^{+/+}$), 11 (sham-*Reg1*$^{-/-}$) or 10 (sTAC-*Reg1*$^{-/-}$) per group. **J,** Regnase-1 (Reg1) protein levels in the hearts (N=7). GAPDH was used as the loading control.

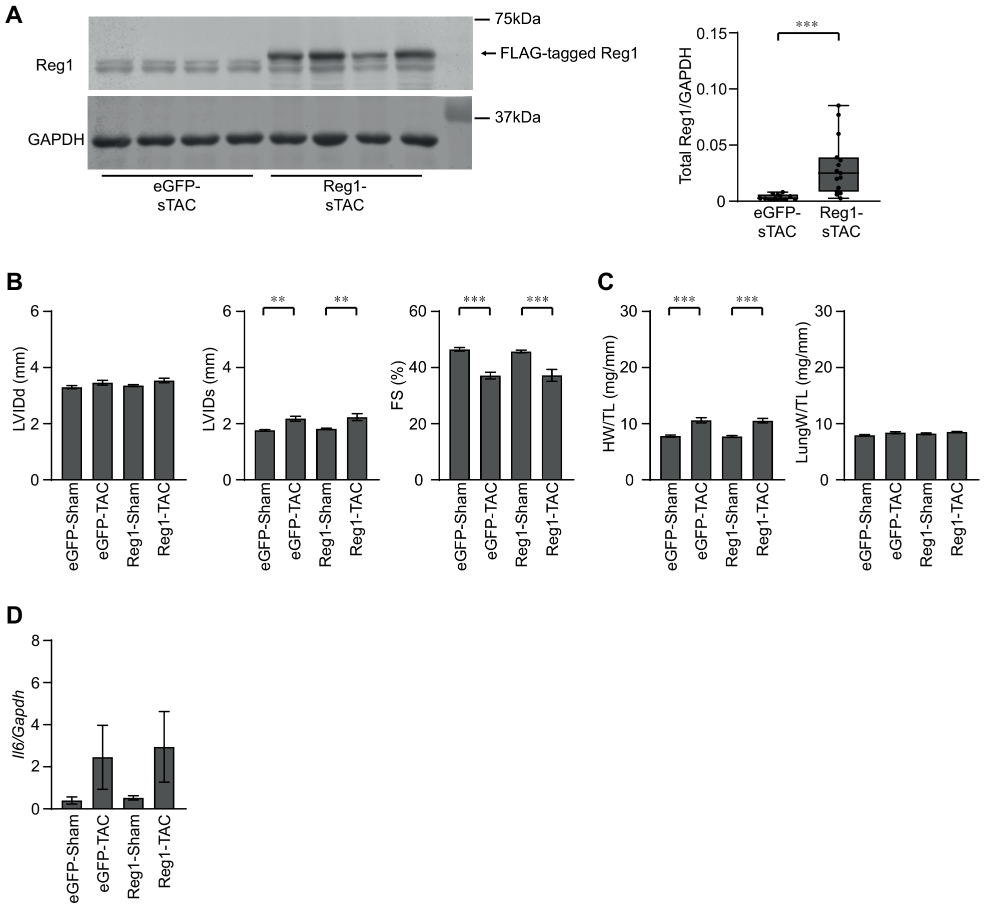

Supplemental Figure IV. Regnase-1 protein in AAV9-infected C57BL/6 mouse hearts subjected to severe TAC and effect of Regnase-1 overexpression on TAC-induced cardiac remodeling.

Mice were analyzed 4 weeks after severe TAC (sTAC). Data are mean ± SEM. $^{**}P< 0.01$, $^{***}P<0.001$. **A,** Regnase-1 (Reg1) protein levels in sTAC-operated hearts infected with eGFP-AAV9 (eGFP-sTAC) or Reg1-AAV9 (Reg1-sTAC). N=11 (eGFP-sTAC) or 15 (Reg1-sTAC). GAPDH was used as the loading control. The right graph shows signal density. **B** and **C**, Wild-type C57BL/6 mice were intraperitoneally injected with AAV9 expressing eGFP (eGFP-AAV9) or Regnase-1 (Reg1-AAV9) and were subjected to TAC 1 week after infection. Sham- or sTAC-operated wild type mice infected with eGFP-AAV9 (eGFP-sham or eGFP-TAC) or Reg1-AAV9 (Reg1-sham or Reg1-TAC) were analyzed 4 weeks after operation. Data were evaluated by one-way ANOVA with the Bonferroni's post hoc test. Data are mean ± SEM. $^{*}P < 0.05$. Echocardiographic (**B**) and physiological (**C**) parameters. N=9 (eGFP-sham), 9 (eGFP-TAC), 9 (Reg1-sham) or 9 (Reg1-TAC). **D**, mRNA expressions of *Il6*. N=4 (eGFP-sham), 4 (eGFP-TAC), 4 (Reg1-sham) or 4 (Reg1-TAC). *Gapdh* mRNA was used as the loading control. The averaged value in eGFP-TAC group was set equal to 1.

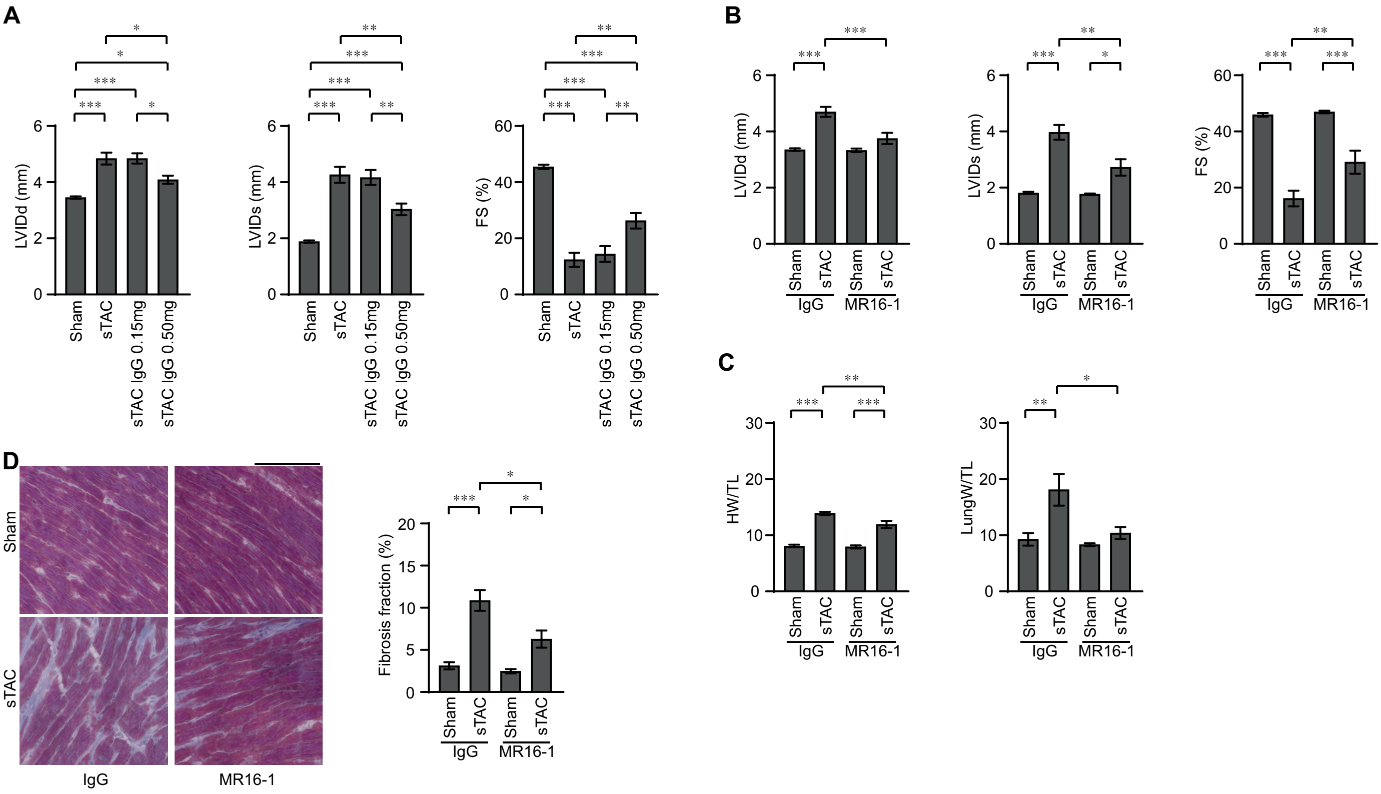

Supplemental Figure V. IL-6 blockade ameliorated severe TAC-induced cardiomyopathy in C57BL/6 mice.

The mice were analyzed 4 weeks after severe TAC (sTAC). Data were evaluated by one-way ANOVA with the Bonferroni's post hoc test. Data are mean ± SEM. $^{*}P<0.05$, $^{**}P< 0.01$, $^{***}P<0.001$. **A,** One week after sTAC operation, wild-type C57BL/6 mice received an intraperitoneal injection of 0, 0.15 or 0.5 mg control IgG once a week. Echocardiographic parameters are shown. N=7 (sham), 6 (sTAC), 7 (sTAC IgG 0.15 mg) or 7 (sTAC IgG 0.5 mg) per group. **B** and **C,** One week after sTAC operation, mice received an intraperitoneal injection of 0.15 mg anti-mouse IL-6 receptor antibody MR16-1 or control IgG once a week. Echocardiographic (**B**) and physiological (**C**) parameters. N=9 (IgG-sham), 9 (IgG-sTAC), 8 (MR16-1-sham) or 9 (MR16-1-sTAC) per group. **D**, Masson's trichrome-stained heart sections. Scale bar, 100 μm. Fibrosis fraction was evaluated. N=4 per group.
